# The Prevalence and Predictors of Digital Proxy Behavior in the United States: Cross-Sectional Survey Study

**DOI:** 10.2196/69806

**Published:** 2025-08-18

**Authors:** Pin Sym Foong, Camellia Zakaria, Pavithren Pakianathan, Andrew Ian-Hong Phua, Gerald CH Koh

**Affiliations:** 1 Telehealth Core, National University of Singapore, 12 Science Drive 2, #10-01, Singapore, 117549, Singapore, 65 6516 4988; 2 Dalla Lana School of Public Health, University of Toronto, Toronto, ON, Canada; 3 Ludwig Boltzmann Institut für digitale Gesundheit und Prävention, Salzburg, Austria; 4 National University Health System, Singapore, Singapore

**Keywords:** digital proxy, caregivers, patients, survey, aging

## Abstract

**Background:**

As dependent adults increasingly require help with managing web-based financial and medical tasks, caregivers often step in to assist—sometimes informally, by sharing login credentials. Despite growing reliance on such digital proxies, limited data exist on the scale of this behavior, who performs it, and how they access accounts. Informal access practices may pose privacy and security risks and increase the potential for elder abuse.

**Objective:**

The aim of this study is to quantify the prevalence of digital proxy behavior in the United States, identify demographic and caregiving predictors of proxy roles, and examine the extent of formal versus informal access methods in medical and financial domains.

**Methods:**

A nationally representative survey (n=657) was conducted among US adults from December 2022 to February 2023. The survey assessed the participants’ life experience of digital proxy behavior, physical caregiving, demographics, and methods and motivations for accessing others’ digital accounts. Logistic regression models were used to analyze predictors of financial and medical digital proxy behavior separately.

**Results:**

In the United States, digital proxy behavior is prevalent and often driven by usability challenges rather than the care recipient’s physical or cognitive limitations. Roughly 49% of respondents reported having had digital proxy duties, with 59% assisting in both medical and financial domains. Predictors of being a digital proxy included being younger, male, more educated, and providing physical care. Additionally, higher income predicted financial proxy behavior, while having more siblings and being from a majority ethnicity predicted medical proxy behavior. Approximately one-third of digital proxies used informal access methods—translating to about 18 million adults nationally. About 10% of the sample logged on by knowing the account owner’s credentials, and about 7% of the sample used the accounts without the owner’s presence. Older female proxies were more likely to use formal access methods.

**Conclusions:**

Informal access practices are widespread and pose potential risks to vulnerable adults. Although most proxies used formal access when available, a significant portion have used insecure, informal methods. Policymakers and system designers should aim to promote secure, user-friendly proxy features and balance access with monitoring mechanisms that balance usability, privacy, and abuse prevention.

## Introduction

The rise in chronic illnesses and an aging global population have placed increasing demands on health care systems, leaving caregivers to fill growing care gaps. In the United States, the economic contribution of unpaid caregivers was valued at approximately $600 billion in 2021, reflecting a 27% increase since 2017 [1]. In aging societies such as the United States, China, and Japan, caregivers frequently support older adults who experience declining cognitive or physical abilities due to aging, illness, or sudden medical events like stroke [2]. These caregiving duties increasingly include managing digital tasks—such as accessing health portals or financial services—which require knowledge of sensitive login credentials [3,4].

Although caregivers may adopt informal digital access methods out of necessity, such practices introduce potential risks, including financial abuse. In the United States, the National Elder Mistreatment Study found that financial abuse by a family member had a 1-year prevalence of 5.2% [5], and financial abuse was involved in 98% of all abusive relationships [6]. The shift toward web-based medical and financial services may exacerbate these risks if proper access mechanisms are lacking. Moreover, even well-intentioned caregivers may overstep boundaries [7]. For example, studies show that some patients do not wish to share all their medical information with caregivers [8], and caregivers may selectively report information to health professionals [9].

Formalized proxy access can improve transparency and reduce misuse by clearly and explicitly authorizing caregivers to act on behalf of care recipients. This access typically involves separate caregiver logins or permissions (eg, joint accounts or caregiver-specific health portal accounts) where activity is trackable, while informal methods—such as sharing usernames and passwords—often lack oversight and accountability.

On the other hand, the literature also suggests that even formal caregiver access raises privacy concerns. Studies show that patients may avoid disclosing sensitive issues if a proxy has full access [8,10]. Although caregivers recognize the benefits of access, they also acknowledge discomfort if it includes financial or stigmatized information [11]. These findings highlight the tension between autonomy and necessary caregiving support. Despite the known benefits of formal access, its implementation is inconsistent, requiring policy informed by larger-scale data on who digital proxies are, how they access accounts, and under what conditions.

We now turn to describing the related work in this area. We present current quantitative data on proxy behavior, then consider previously reported behaviors that promote and challenge proxy behavior.

Prior research in human computer-interaction has examined digital proxy behavior using mostly qualitative methods such as in a previous study by Hunsaker et al [12]. Where quantitative method have been used, the data is not nationally represenative. For example, a Canadian study by Latulipe et al [13] involving 42 participants found that 28% had formal proxy access, yet 59% knew the care recipient’s login credentials, raising concerns about misuse. A US-based survey study showed that 45% of hospital staff supported password sharing between patients and caregivers [14]. Existing national-level surveys tend to focus on general caregiving intensity or caregiver use of digital tools [2,15,16], rather than the access behaviors involved.

However, a comprehensive understanding of the scale of digital proxy behavior—particularly informal proxying—remains absent, leading to wide deviations in reported prevalence. For example, surveys examining patient behavior showed that most patients (79%) were open to sharing access to personal health records with kin, most commonly a spouse, child, or family member [17]. On the other hand, going by the estimates of older adults with medical directives, the prevalence of proxies is at about one-third of the US population [18]. In contrast, in Germany, 64.6% had assigned medical powers of attorney—mostly to adult children or grandchildren [10].

Beyond prevalence, knowledge is also incomplete regarding the factors that influence who becomes a digital proxy. Qualitative studies suggest that younger individuals and those closely related to the care recipient are more likely to help with digital tasks [19-21]. Others note that even non-kin or older adults can be involved [12,22] and that the eldest children in families often act on behalf of the care recipient [23]. One small quantitative study found that younger users with high technological self-efficacy were more likely to offer digital help, but factors like income, gender, and education were not strong predictors [24].

In terms of barriers, low usability of health portals is consistently cited. Quinn et al [25] found that both patients and caregivers struggled with poorly designed portals, suggesting that training and design improvements are needed. Wolff et al [26] note that while the Health Insurance Portability and Accountability Act permits reasonable information sharing with caregivers, security protocols can make access cumbersome—particularly for older users [27].

In summary, although digital proxying is a common and growing practice, there is little national data on who engages in it and how. Usability challenges frequently motivate informal proxy behavior, and proxies are often younger, technologically savvy kin. This study offers the first nationally representative picture of digital proxy behavior in the United States, clarifying the scale, access methods, and predictors in medical and financial domains—two areas where decision-making capacity is often transferred to caregivers [28,29]. By distinguishing between formal and informal proxying, this research provides critical insights for the development of safer, more inclusive systems that empower caregivers while protecting vulnerable users. We sought to answer three key questions: (1) What is the prevalence of digital proxies? (2) What factors predict the likelihood of someone acting as a proxy? and (3) How common are informal methods when carrying out their digital proxy duties?

The rest of the paper is divided as follows: We first describe methods, explaining the design of the survey and data collected and processed prior to our analysis. We then report the results of our analyses in two parts. First, we discuss the demographic and caregiving factors significant to digital proxies, and second, we delve into the informal and formal digital practices engaged by these proxies. Finally, we discuss the need to protect care recipients while accommodating caregiving realities.

## Methods

### Overview

We conducted a survey of adults on financial proxies, medical proxies, and medical decision-making with 107 questions in the United States. It included all demographic data and items relevant to digital finance and medical proxies. The current analysis excluded survey items on medical decision-making. The entire survey is available in Multimedia Appendix 1. The study was reviewed by the institutional review board of the National University of Singapore, with approval number NUS-IRB-2022‐412. The Checklist for Reporting Results of Internet E-Surveys (CHERRIES) can be seen in Checklist 1.

We designed the survey questions to capture the lived experience of caregivers over time. We did not take the more common approach to capture a snapshot of care associated with one care recipient, only at the time of the survey. Instead, we focused on gathering information about peak experiences and the experience of caregiving over a period of time. For example, we asked about the highest frequency of physical care supplied and allowed participants to report the methods they have ever used to access accounts. As a result, we phrase outcomes in terms of patterns of behavior, rather than instances of behavior—“participants who have ever had higher physical care duties are more likely to be also having digital care duties.” This approach introduces some limitations, which we discuss in the Limitations section.

### Ethical Considerations

The study was reviewed by the institutional review board of the National University of Singapore, with approval number NUS-IRB-2022‐412. Participants indicated consent via a web-based form prior to the survey. The consent did not include consent for secondary analysis. We preserved the privacy and confidentiality of the participants by anonymizing the survey at source, with no personally identifiable information being collected. Participants were compensated by Qualtrics, the survey panel company. They compensated the respondents with either cash, vouchers, or coupons.

In the next section, we describe the definitions of the terms used, the survey design, and sources of the items we used, where available.

### Language and Definitions

In this survey, we conceived of a *proxy* as someone who carries out tasks on behalf of another adult. We divided the tasks into physical and digital tasks.

**Figure 1.**
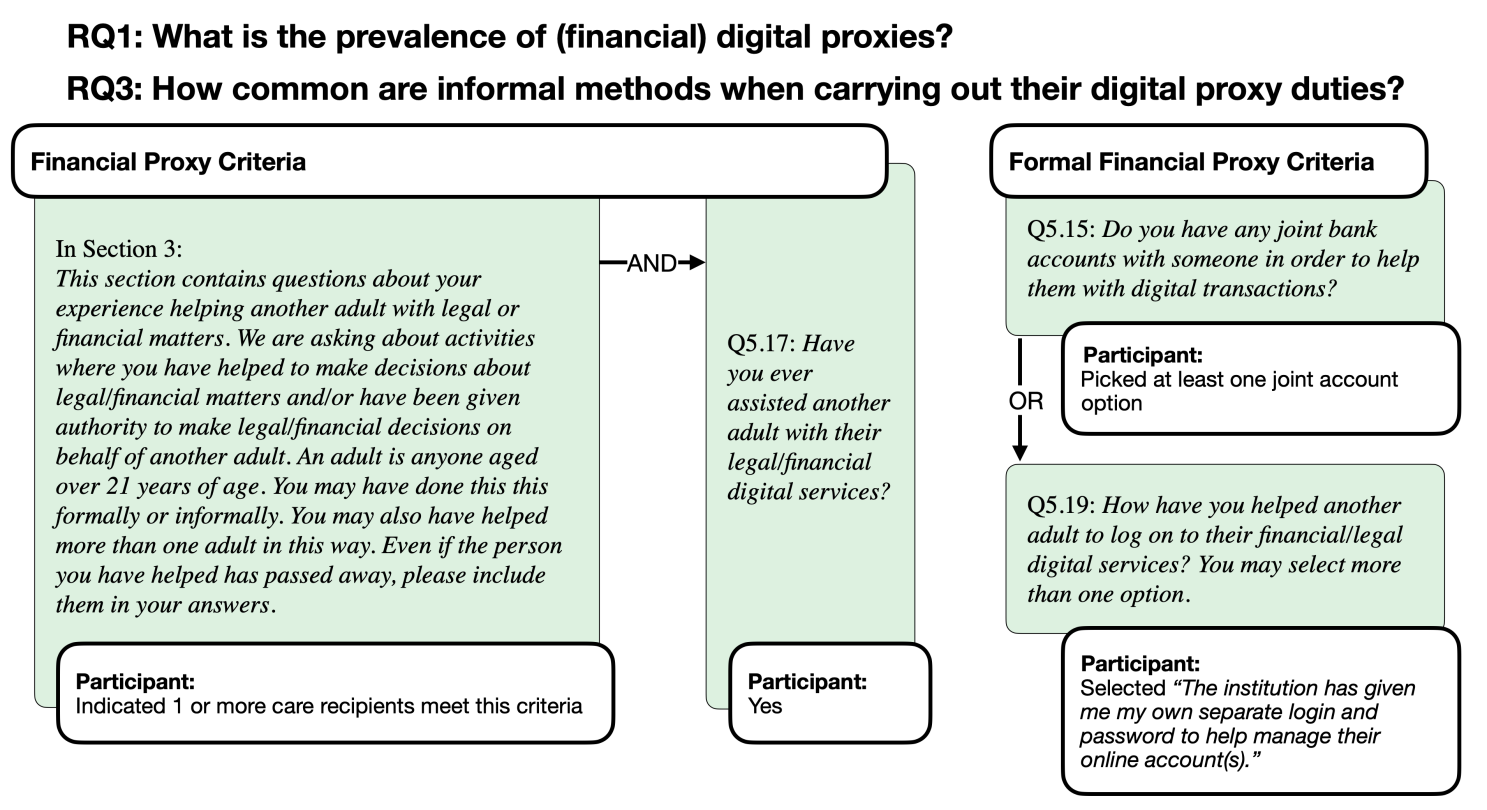
Research questions regarding financial digital proxies and formal access and the survey items that were used to identify them.

**Figure 2.**
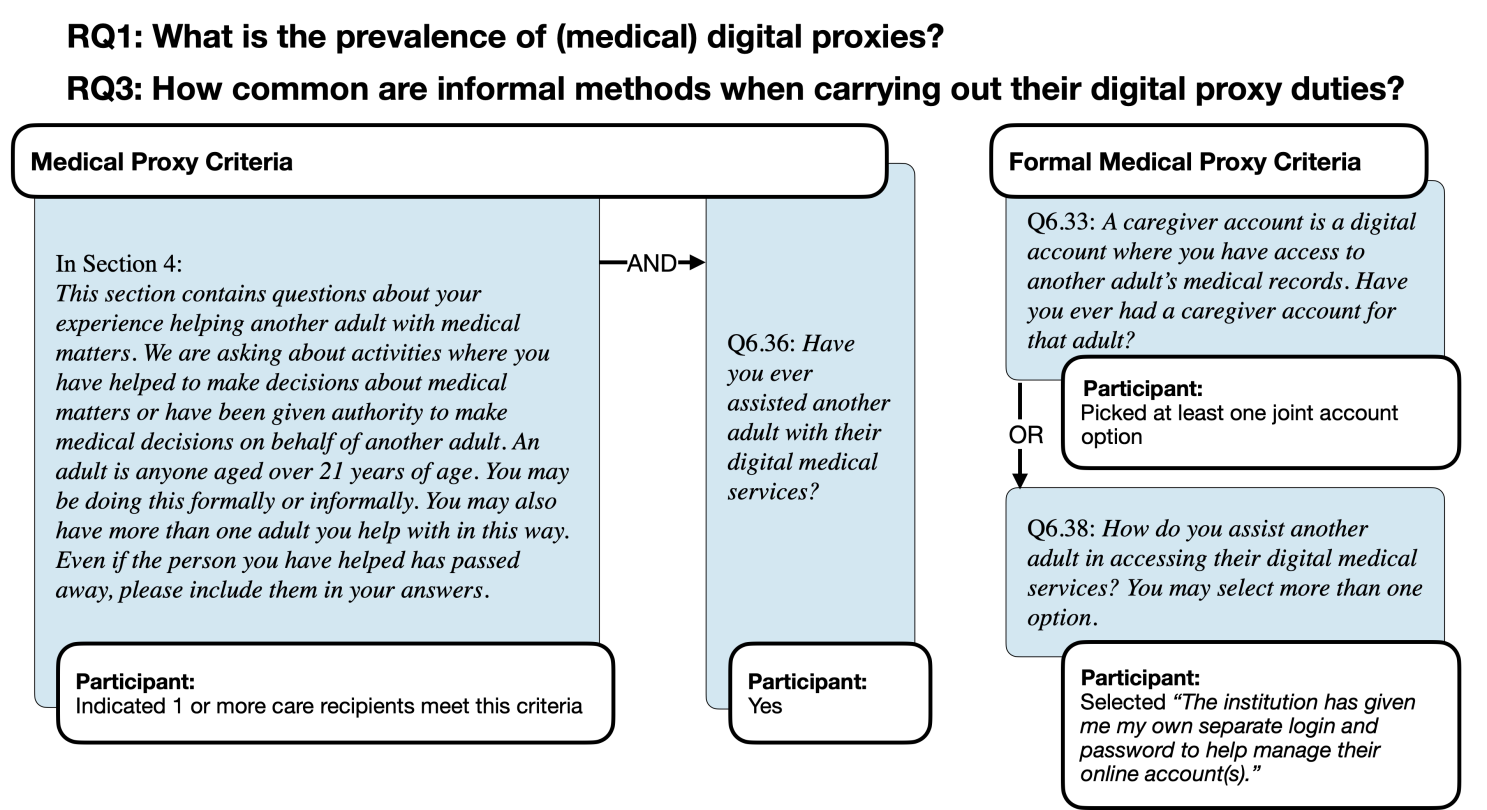
Research questions regarding medical digital proxies and formal access and the survey items that were used to identify them.

In this paper, we use the term *delegator* to refer to the adult who requires help with these tasks, but in the survey, we used the simpler language of “helping another adult” to refer to the delegator (see Figure 1 Figure 1 and , Figure 2). Additionally, we used this phrasing because we wanted participants to distinguish helping behavior from parenting duties where a person below 21 years old is helped.

We define *formal access* using the concept of delegation of authority in role-based access control [30,31] models. Hence, when a caregiver has formal access, they are authorized to perform specific actions on behalf of the care recipient (eg, through a proxy caregiver account in the MyUPMC app [32]). On the other hand, the National Institute of Standards and Technology defines unauthorized access as the act of gaining logical or physical access without official permission to a network, a system, an application, data, or another resource [33]. Throughout this paper, we use the term *informal access* to describe these methods that are not authorized by the organization, such as sharing credentials or using the account on behalf of the care recipient.

Table 1 shows the types of demographic data that we surveyed and the reasons for inclusion.

**Table 1.** Demographic items included in the survey based on existing literature about possible predictors.^a^

Survey items	Reason for inclusion
Demographics (age, gender, education, income, housing, siblings, birth order, employment, marital status, ethnicity)	Age and gender were previously reported as relevant Regarding siblings and birth order, Ong et al [23] suggested some influence of being the oldest sibling Education and income were previously associated with financial literacy The remaining demographics were included for complete reporting
Support for Instrumental Activities of Daily Living, Support for Activities of Daily Living	Physical caregiving is a common measure of caregiver behaviors. We want to contextualize digital caregiver behaviors against physical caregiving.

a The items are related to research question 2: What factors predict the likelihood of being a digital proxy?

### Survey Design

The demographics collected are summarized in Table 2. We included predictors previously identified in the literature such as *income*, *ethnicity*, *employment*, *education*, *housing*, *age*, *marital status*, *birth order*, and *gender*.

**Table 2.** Summary of 657 verified participants’ demographics, gender, marital status, age, housing, income, siblings, ethnicity, employment, and educational attainment.

Participants	675 total, 657 verified US nationals/residents over multiple rounds of collection between December 2022 and February 2023
Gender	Female (332), male (325)
Marital status	Married (266); unmarried (262); widowed, divorced, separated (129)
Age (years)	21‐24 (56), 25‐29 (47), 30‐34 (77), 35‐39 (63), 40‐44 (62), 45‐49 (65), 50‐54 (56), 55‐59 (49), 60‐64 (56), 65‐69 (63), >70 (63)
Housing	Single family home (435), 2 or more units (177), mobile/trailer homes (45)
Income (US $)	<30,000 (193), 30,000‐49,000 (150), 50,000‐99,000 (197), 100,000‐159,000 (74), 150,000‐199,000 (28), >200,000 (15)
Number of siblings	0 (84), 1 (176), 2 (163), 3 (99), 4 or more (135)
Ethnicity	White (418), Black (107), mixed (40), Hispanic/Latino (42), Asian (36), Native American (8), others (6)
Employment	Employed: full-time (279), part-time (87), self-employed (7); unemployed: retired (141), disabled (20), unemployed (119), student (4)
Education	Degree and above (300), high school (329), below high school (28)

**Figure 3.**
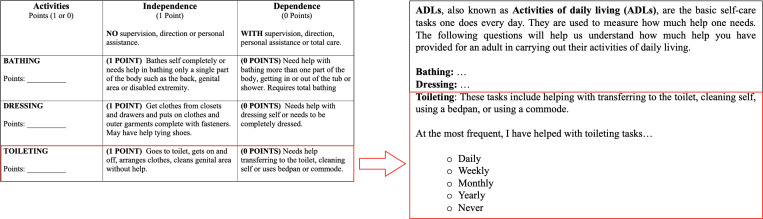
The left side of the figure shows the actual scale in the Katz Index of Independence in ADL [35], which is adapted in our survey instrument (right side of the figure) to capture the frequency at which caregivers are involved with helping another adult in ADL. ADL: Activities of Daily Living.

We adapted two functional assessment scales to assess the prevalence of physical helping behaviors. The first is the Instrumental Activities of Daily Living (IADL) scale, which measures function in 8 areas of independent living, such as driving, housekeeping, and laundry [34]. The second scale is the Activities of Daily Living (ADL) scale. There are 6 ADL in the scale (washing, dressing, feeding, toileting, walking/moving, transferring) that make up independent living [35]. In both scales, the absence of any of these activities is used to measure disability in the adult being helped. In adapting these scales for participants of this survey, we framed the question to first describe the task, then ask what the highest frequency is of help the participants have offered (Figure 3). The options ranged from *daily*, *weekly*, *monthly*, *yearly*, to *never*. The intention was to capture the highest reported intensity of helping behavior in a person’s lifetime.

We asked about financial digital helping behaviors in the next block of items in . Participants were asked if they had financial proxy duties using any variation of a joint account from a bank. Then, we asked if participants had ever helped another adult with digital financial accounts. Finally, we asked about the method of access, ways of helping with use, and the reasons for helping with access. We reviewed studies on potential barriers to adopting financial, legal, medical, and digital services and extracted possible options [36-44].

To identify the methods of helping with login, we asked: *How have you helped another adult to log on to their financial/legal digital services? You may select more than one option*.

- *I know the username and password for their online account(s*)
- *The adult logs in with a username and password and I help them (I don’t know the username and password*)
- *The adult uses their fingerprint (or other biometrics) to log in*
- *The institution has given me my own separate login and password to help manage manage their online account(s*)

To identify the methods of helping with using the digital services, we asked: *How have you helped another adult use their financial/legal digital services online? You may select more than one option*.

- *I offer some help, but the person does it mostly independently*
- *I provide ongoing assistance while next to the person*
- *I use the person’s account on their behalf, while they are present*
- *I use the person’s account on their behalf, even when they are not present*

To identify the reasons for helping with access, we asked: *Provide the reason(s) why you think your help was needed to manage a digital service for another adult. You may choose more than one answer*.

- *The app is not user friendly for them*
- *They are unfamiliar with using a technology device to access the app*.
- *They do not have a device or equipment to access their digital accounts*.
- *They have cognitive limitations (eg, existing conditions and cognitive decline with age) that constrain their ability to use the app*.
- *They have concerns about sharing their data with third parties*.
- *They have physical limitations (eg, poor vision and poor motor skills) that constrain their ability to use the app*.
- *They see using the app as a difficult task that should be avoided*.

In the last block of items, we asked about medical digital helping behaviors. The participants were asked if they had a caregiver account for assisting with medical matters. Then, we went on to ask if participants had ever helped another adult with digital medical accounts (). Finally, we asked about the method of access and their perceived reasons they needed to proxy, as listed above.

Survey items were not randomized, and adaptive questioning was used to make the survey less complex for respondents. Completeness checks were built into the survey instrument. Participants could review and change their responses with the review function.

Tables 5 and 6 show how the participants were classified as having no digital proxy duties, informal digital proxy duties, and formal digital proxy duties for financial and medical domains, respectively. Note that respondents who answered “Unsure” were classified as “No” in our sample to increase our certainty that formal proxies have a clear report of formal access. In the sample, 189 participants were both financial and medical digital proxies, while 72 were financial (only) and 59 were medical (only) digital proxies.

**Table 3.** Questions and responses identifying participants who had no digital proxy duties, informal duties, and formal digital proxy duties related to financial duties (N=657).

Survey items	Not proxy	Conditions for financial digital proxies			
		Informal proxy behavior	Formal proxy behavior (possible answer combinations)		
Q5.17 *I have helped with digital financial access*	No	Yes	Yes	Yes	Yes
Q5.15 *I have a joint account*		No	Yes	No	Yes
Q5.19 *I have my own separate account*		No	Yes	Yes	No
Total, n (%)	396 (60.3)	89 (13.5)	172 (26.2)		

**Table 4.** Questions and responses identifying participants who had no digital proxy duties, informal duties, and formal digital proxy duties related to medical duties (N=657).

Survey items	Not proxy	Conditions for medical digital proxies			
		Informal proxy behavior	Formal proxy behavior (possible answer combinations)		
Q6.36 *I have assisted another adult with digital medical services*	No	Yes	Yes	Yes	Yes
Q6.33 *I have a digital caregiver account*		No	Yes	No	Yes
Q6.38 *I have my own separate account*		No	Yes	Yes	No
Total, n (%)	409 (60.3)	89 (13.5)	159 (24.2)		

### Recruitment

The survey was sent to Qualtrics, a panel survey company that was tasked with recruiting a nationally representative sample of US respondents from December 2022 to February 2023. The company invites respondents by email (indicating research purpose, incentives, and duration) or through advertisements on the respondents’ login panel. To avoid self-selection bis, the survey invitations are kept general without specific details about the survey. The company compensated the respondents with either cash, vouchers, or coupons. Exclusion criteria were participants who were not residents of the United States and were aged below 21 years at the time of the survey. Attention checks such as “*Do you commit to providing thoughtful answers to the questions throughout the survey?*” were built in to ensure response robustness. Cookies were used on the last page, valid for a week, with duplicate entries avoided by preventing users with the same IP address access to the survey twice. A manual log file was also checked for multiple entries. No minimal timestamp was used—we grouped related items across multiple pages and mixed mandatory and optional items to maintain engagement. Residency was checked by verification performed by our research team, where we excluded responses with embedded data that failed the quality check for IP addresses and the participant’s self-report.

### Participant Summary

Our sample participants were nationally representative. We established this by comparing the various parameters (income, age, ethnicity, and housing) to the data from the most recent national census data by the US Census Bureau from 2020 [45], as per Multimedia Appendix 1. Note that the US census data percentage is reported as is and may not add up to 100%.

Table 2 summarizes the key details of our data collection. The participation rate was 64%. The completion rate was 77% and 675 participants completed the survey, with 657 respondents passing verification tests. Our analysis, moving forward, accounts for these 657 respondents. The average time these participants took to complete the survey was 18.24 minutes.

### Scoring Procedure

#### Frequency of Physical Care Assistance

In adapting the IADL and ADL functional assessment scales, our scores were on a 0‐4 Likert scale (0=never, = yearly, 2=monthly, 3=weekly, 4=daily). We normalized the sum of the frequency of all IADL and ADL components to a range of 0‐1, where 0 denotes a person never assisting in any key life tasks of another adult, and 1 denotes a person assisting in all aspects of IADL or ADL daily.

#### Digital Proxy Behavior

In , we demonstrated how we separated formal and informal proxies. We classified formal proxies as participants who reported having had any kind of formal access, such as having a joint account, having a digital caregiver account, and/or having their “own separate account.”

## Results

### Prevalence of Digital Proxy Behavior

In our sample, the total unique number of participants reporting digital proxy behavior was 320 of 657. Of these, 261 were classified as financial digital proxies (Table 3), and 248 were classified as medical digital proxies (Table 4). These groups had a substantial overlap, with 189 (59%) reporting both financial and medical digital proxy tasks.

We further subdivided the respondents into those who reported using formal versus informal access methods. Overall, 89 of 657 participants reported that they have been informal financial proxies (Table 3), and the number was coincidentally the same for informal medical proxies (Table 4). Of these, 32 informal proxies have had both duties, making a total of 146 unique informal digital proxies, which represented 22.2% of our population sample.

**Figure 4.**
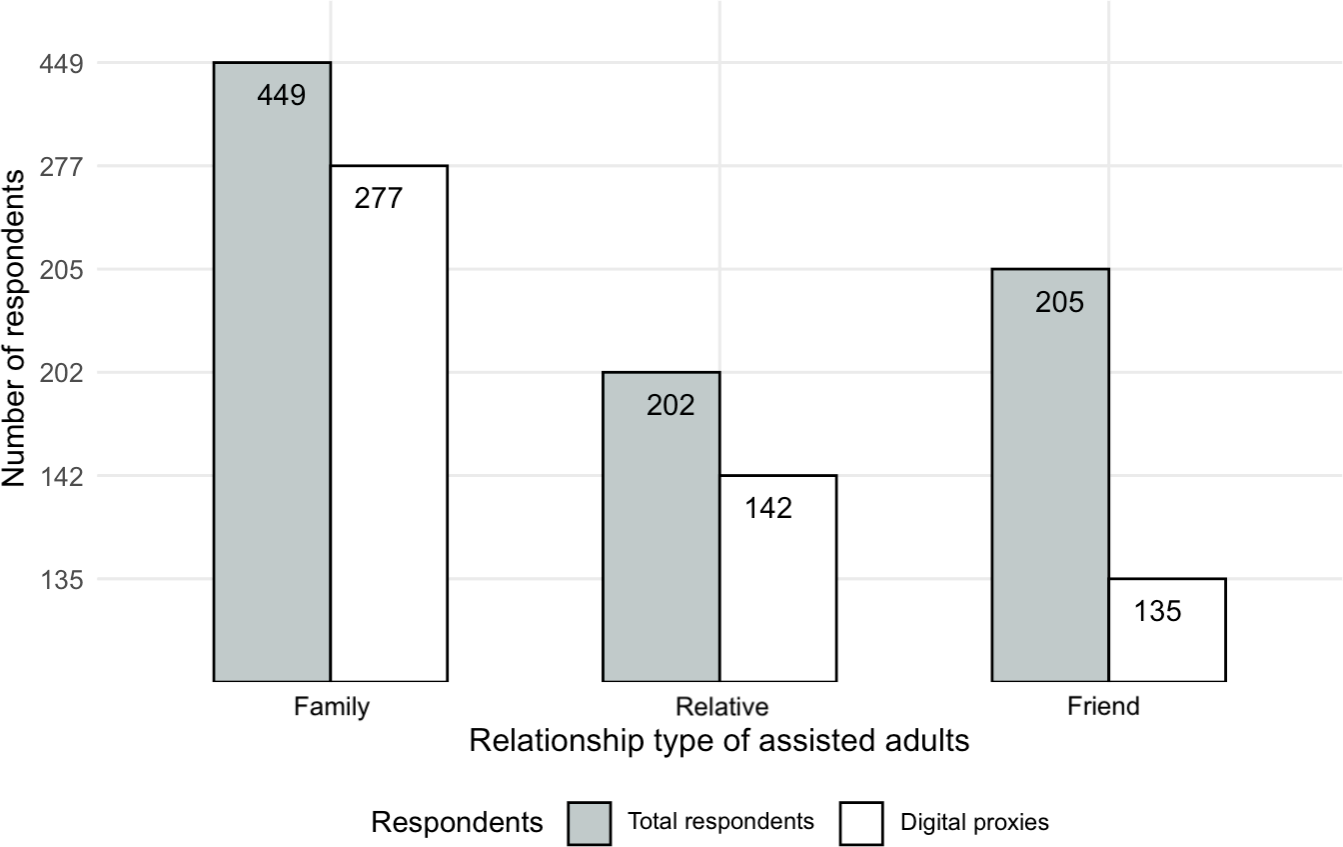
Counts of digital helping in the population, categorized by reported relationship to the person who is being helped. The total is larger than 320 (unique digital proxies) as respondents could select more than one option.

Next, we were interested in understanding who the delegators were (relative to the proxy). As plotted in Figure 4, of all 657 respondents, 68% (n=449) reported ever helping an immediate family member, and approximately 30% supported a relative (n=202) or a friend (n=205). We observed that digital proxy behavior was mainly for helping immediate family and relatives, whereas physical helping behavior also extended to helping friends. For example, digital proxies made up 62% (227/449) of the respondents who helped with physical care matters for an immediate family member.

The rest of this paper investigates this subsample of digital proxies (financial digital proxies n=261; medical digital proxies n=248), uncovering tension points in digital proxies to promote safe practice.

### Modeling Types of Digital Proxies

We used generalized linear models to investigate the relationship between participants’ demographic factors and physical care behavior (independent variables) in taking up proxy duties related to financial and medical matters (dependent variables) for their delegator. This model choice is due to many predictors not being normally distributed and a mix of continuous and discrete values.

**Figure 5.**
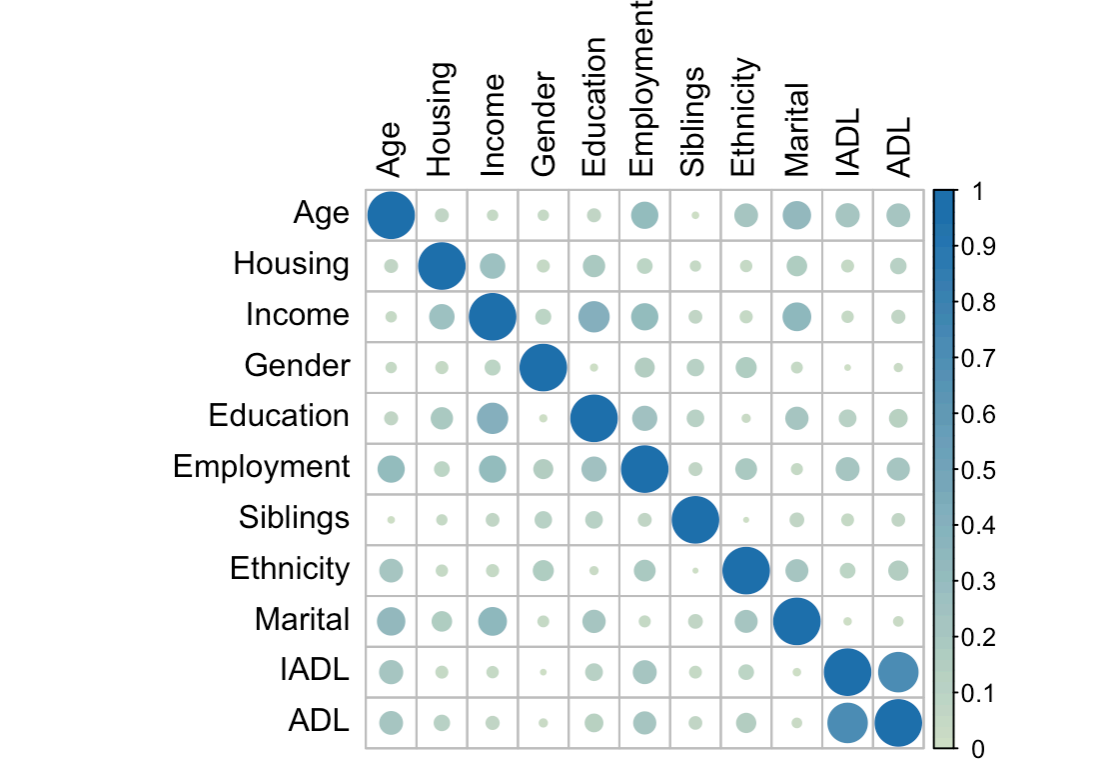
Correlation matrix of predictors showing no correlation higher than *r*=0.71*.* ADL: Activities of Daily Living; IADL: Instrumental Activities of Daily Living.

Our model specifies the logit link between both sets of variables to clarify how the combination of IVs as linear predictors relates to participants’ response values in being a proxy (*Yes=1* or *No=0*), for two types of digital duties, as per Table 3 (financial) and Table 4 (medical). Figure 5 shows the correlations between all predictors, with all correlations no more than *r*=0.71. For this reason, we considered all variables in our model.

#### Goodness of Fit

We performed step-wise regression to procedurally determine the best combination of predictors and verify the goodness of fit for both models. Performing a backward selection resulted in a simplified model with the lowest Akaike information criterion (AIC). The lowest AIC value quantifies information loss due to model simplification without compromising performance. A lower value emphasizes a lesser tendency for the model to overfit our data.

In measuring the goodness of fit, unlike linear regression in which *R*^2^ represents the proportion of variance explained by predictors in a model, there is no agreed-upon measure in logistic regression analysis analogous to *R*^2^. Still, several competing measures of pseudo-*R*^2^ exist. We calculated McFadden pseudo-*R*^2^ to measure how well the model fits.

Defined as $pR_{MF}^{2}=1-(df_{residual}/df_{null}),\;df_{residual}$ denotes the maximized likelihood value from the current model that includes all predictors and $df_{null}$ denotes the maximized likelihood value from the model with only an intercept and no covariates. A good model should produce a log-likelihood close to 0 since the ratio of the two log-likelihoods,

*$df_{residual}$* and $df_{null}$, will be close to 1. Values of 0.2 to 0.4 for $pR_{MF}^{2}$ represent excellent model fit [46].

Table 5 provides the model summary for predicting each type of digital proxy duty. Simplifying our models to only selected predictors reduced AIC to 683.58 (from 690.9 in the finance digital proxy model) and 666.73 (from 668.13 in the medical digital proxy model). The *pR^2^* for the financial and medical models were 0.25 and 0.27, respectively, indicating excellent model fit. Further, calculating the chi-square statistic, *χ^2^*, for both models, resulted in *P*<.001. The coefficient estimates for these models are summarized in Tables6  7.

**Table 5.** Model summary for financial and medical digital proxy, showing similarities and differences in the predicting factors.

Model	Physical care	Demographics	AIC^a^	Deviances (*df*)	*pR^2^*	Chi-square	*P* value
Financial digital proxy	IADL^b^, ADL^c^	Age; income; gender: male; education; marital: married, unmarried	683.58	Null: 882.86 (656); residual: 665.58 (648)	0.25	217.28	<.001
Medical digital proxy	IADL, ADL	Age; gender: male; education; employment: unemployed; siblings; ethnicity: underrepresented; marital: married, unmarried	666.73	Null: 870.94 (656); residual: 644.73 (646)	0.27	226.21	<.001

a AIC: Akaike information criterion.

b IADL: Instrumental Activities of Daily Living.

c ADL: Activities of Daily Living.

**Table 6.** Coefficient summary to predict a proxy undertaking financial digital duties.

Coefficient	Estimate	95% CI	Standard error	*z*-score	*P* value
(Intercept)	−4.25	−5.54 to −3.02	0.64	−6.63	<.001
Age	−0.07	−0.14 to −0.00	0.04	−2.06	.04
Income	0.18	0.01 to 0.36	0.09	2.08	.04
Gender: male	0.57	0.19 to 0.96	0.19	2.94	.003
Education	0.36	0.00 to 0.72	0.18	1.96	.05
Marital: married	0.18	−0.36 to 0.73	0.28	0.66	.51
Marital: unmarried	−0.32	−0.91 to 0.27	0.30	−1.06	.29
Instrumental Activities of Daily Living	3.49	2.51 to 4.53	0.52	6.78	<.001
Activities of Daily Living	0.82	0.20 to 1.45	0.32	2.59	.009

**Table 7.** Coefficient summary to predict a proxy undertaking medical digital duties.

Coefficient	Estimate	95% CI	Standard error	*z*-score	*P* value
(Intercept)	−3.77	−5.21 to −2.38	0.72	−5.22	<.001
Age	−0.13	−0.21 to −0.05	0.04	−3.36	.001
Gender: male	0.61	0.22 to 1.01	0.20	3.03	.002
Education	0.42	0.06 to 0.78	0.18	2.30	.02
Employment: unemployed	−0.34	−0.78 to 0.09	0.22	−1.56	.12
Siblings	0.22	0.07 to 0.37	0.08	2.84	.004
Ethnicity: underrepresented	−0.47	−0.90 to −0.04	0.22	−2.12	.03
Marital: married	0.13	−0.42 to 0.68	0.28	0.47	.64
Marital: unmarried	−0.46	−1.07 to 0.16	0.31	−1.46	.14
Instrumental Activities of Daily Living	2.69	1.69 to 3.75	0.53	5.13	<.001
Activities of Daily Living	1.39	0.74 to 2.04	0.33	4.18	<.001

### Demographic Profile

**Figure 6.**
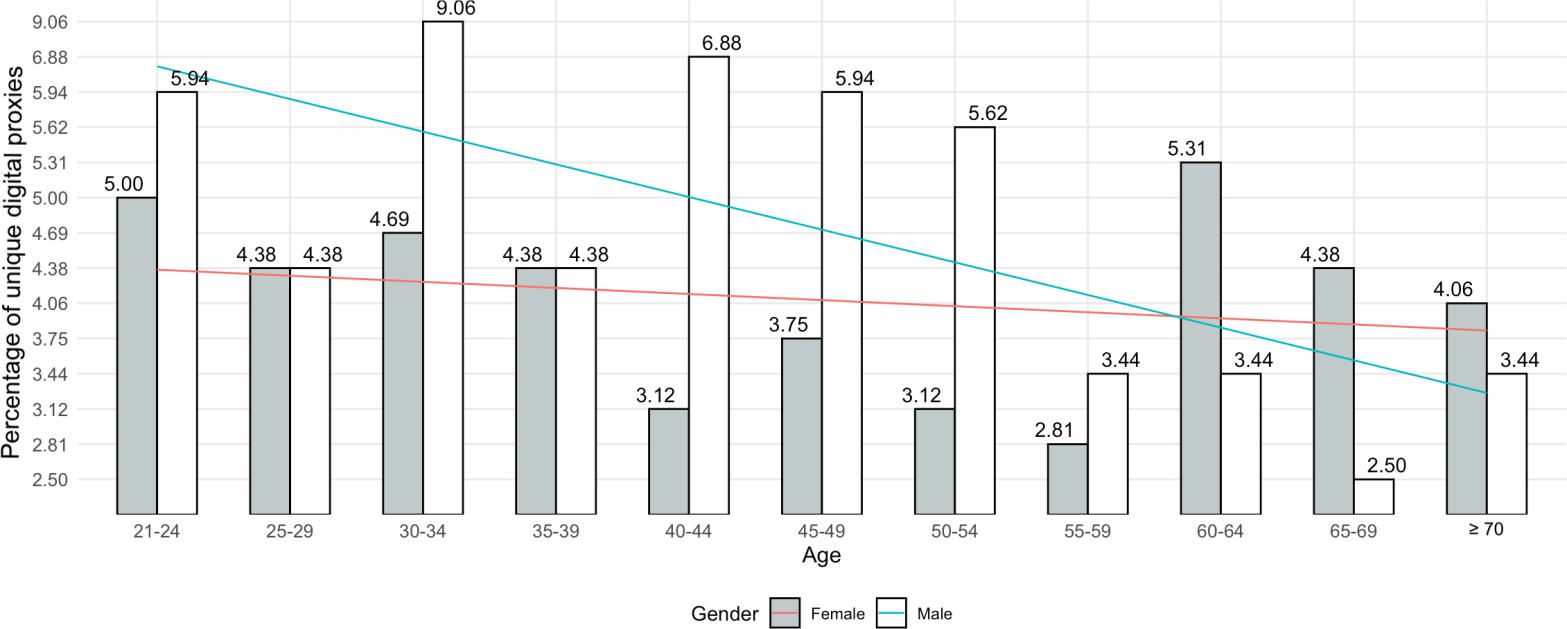
Bar chart plotting the percentages of unique digital proxies by age and gender. The chart shows a preponderance of younger, male adults reporting digital proxy behavior. However, the trend line suggests an increase in female digital proxies above age 60.

Our results yielded *age*, *gender*, and *education* as common demographic factors across finance and medical digital proxies at *P*≤.05. To delve deeper into this predictor, we plotted the distribution of unique digital proxies (n=320) across *age* and *gender* (Figure 6). Overall, we observed a majority of (finance and medical) digital proxies as male respondents; however, there is a decreasing participation trend among those over 55 years old. The log odds of a younger person and a male assisting with financial digital matters increased by 0.07 (age) and 0.57 (gender), respectively. Similarly, a younger person of the male gender was more likely to take up medical digital duties, with a 0.13 (age) and 0.61 (gender) increase in log odds. Interestingly, as per Figure 6, this situation is reversed for digital proxies above 60 years old, with a predominance of female digital proxies.

**Figure 7.**
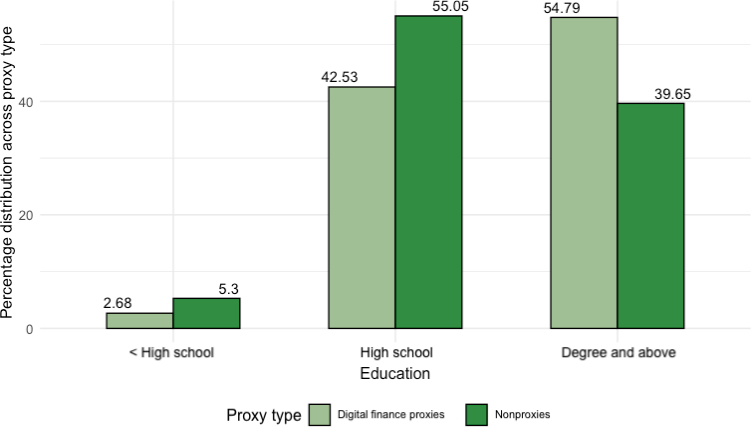
Bar chart of all respondents (n=657) comparing percentage of highest educational attainment for financial digital proxies versus nonfinancial digital proxies, normalized across proxy type, showing most proxies attaining higher education.

**Figure 8.**
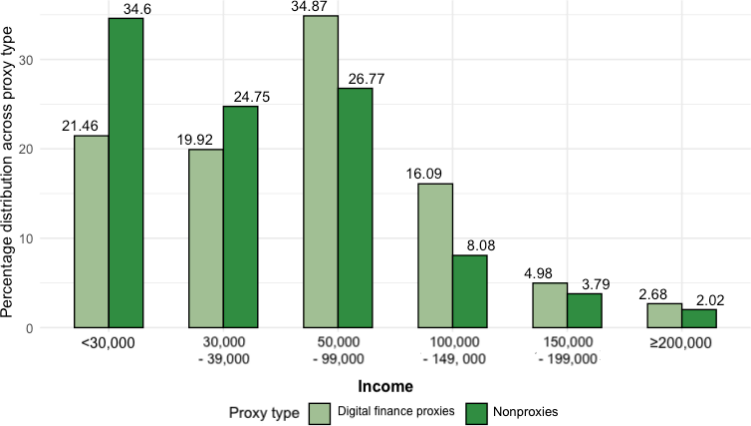
Bar chart of all respondents (n=657), showing percentage of household income distribution among financial digital proxies versus nonproxies, showing most nonfinancial digital proxies coming from lower-income households.

In terms of *education*, as per Figure 7, most finance digital proxies attained a college degree. We observed a similar trend among medical digital proxies, where 54% (n=134) were college-educated. For a one-unit increase in one’s education bracket, the log odds of having assisted with finance and medical digital duties increased by 0.18 and 0.42, respectively. *Income* was additionally a significant predictor for finance digital duties at *P*<.05. As per Figure 8, most proxies earned within the range of $50,000-$99,000, which includes the national average of about $70,000 per household [45]. Specifically, the log odds of reporting assisting in financial digital duties increased by 0.36 for a one-unit increase in one’s income and education bracket. In contrast, most participants who had no experience with finance digital proxies reported a household income threshold of less than $30,000 annually.

**Figure 9.**
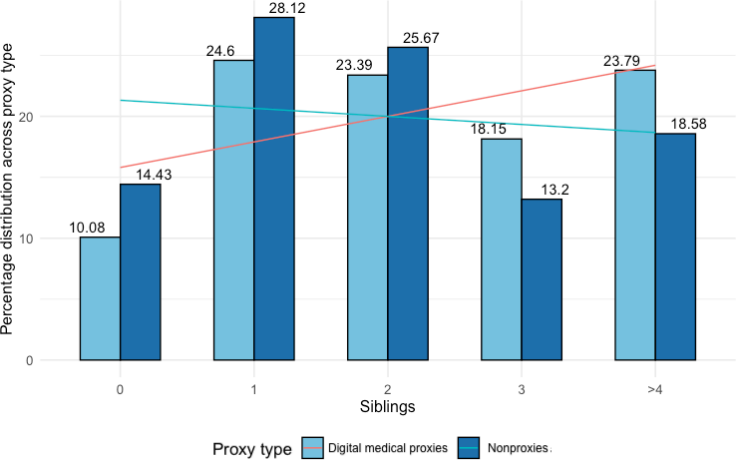
Bar chart of all respondents (n=657) showing the distribution of the number of siblings among medical digital proxies and nonproxies, normalized across proxy type.

**Figure 10.**
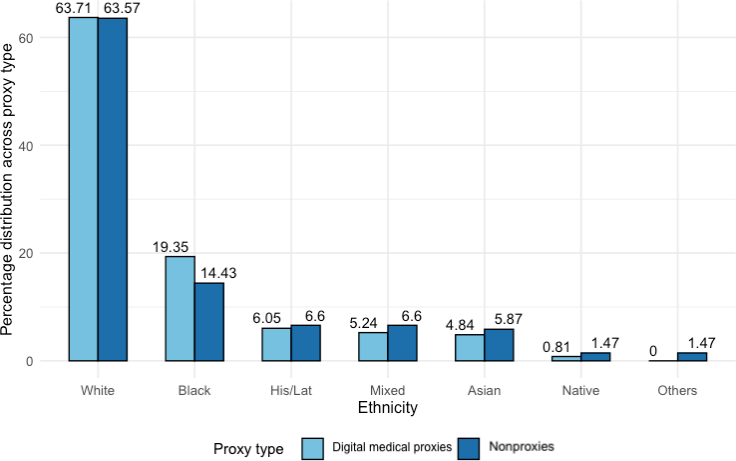
Bar chart of all respondents (n=657) showing the distribution in ethnic groups among medical digital proxies and nonproxies, normalized across proxy type. His/Lat: Hispanic/Latino.

Additional significant predictors relevant in determining having experience as medical digital proxies included the number of *sibling*(s) a person has and *ethnicity*. As per Figure 9, we observed an increasing trend of medical digital proxies with more siblings. For every one-unit change in the number of siblings, the log odds of a person assisting with medical digital duties increased by 0.22. Black, Native, Asian, Latino, Hispanic, and mixed ethnic groups made up the minority (n=239, 36%) of medical proxies (Figure 10). Although we observed negligible differences in the percentage distribution of ethnically White respondents ever taking up medical digital duties, the underrepresented communities seemed less likely to have ever taken up medical digital proxy duties, with log odds decreasing by 0.47. Figure 10 suggests that there may be some variation among underrepresented ethnic groups, but the differences between proxies and nonproxies were not significant (*P*>.50).

### Involvement With Key Life Tasks

Recall that our scoring in the frequency of IADL and ADL assistance spans from 0 to 1, where 0 denotes a person never assisting in any key life tasks of another adult and 1 denotes a person assisting in all aspects of IADL or ADL daily.

**Figure 11.**
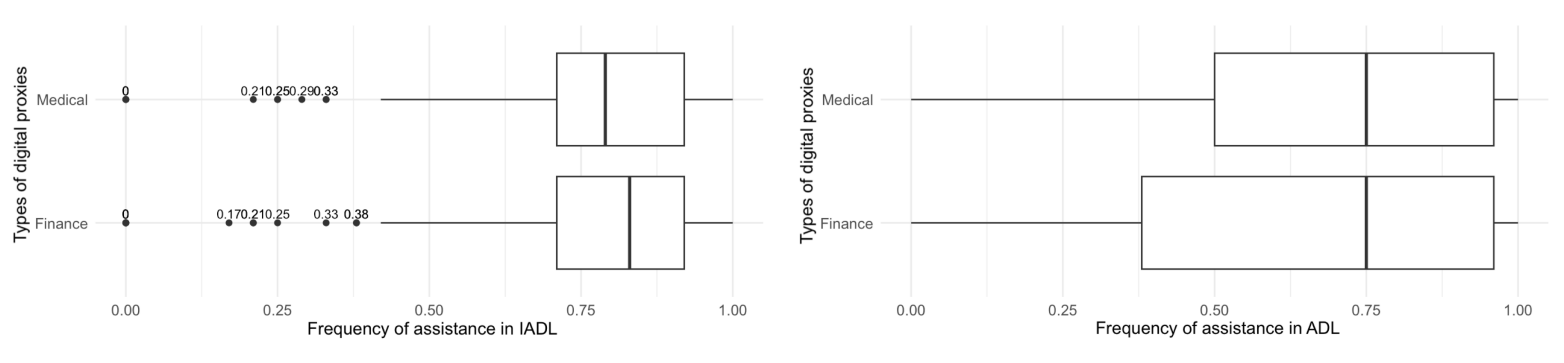
Distribution of IADL and ADL assistance score among digital proxies. ADL: Activities of Daily Living; IADL: Instrumental Activities of Daily Living.

Figure 11 compares involvement in two types of key life tasks, IADL and ADL, among finance and medical digital proxies. The median frequency of IADL assistance was 0.83 among finance (mean 0.77, SD 0.19) and 0.79 among medical (mean 0.77, SD 0.18) digital proxies. In contrast, the distribution of ADL assistance scores spanned a broader range, with the median frequency equal to 0.75 among both digital proxies (finance: mean 0.65, SD 0.34; medical: mean 0.67, SD 0.32). Note that we did not remove outliers as they represent natural variations in the population.

IADL and ADL are significant predictors of finance and medical digital proxies, achieving *P*<.01 significance, that is to say, the more a person had previously experienced assisting another adult with their physical care activities (IADL and ADL), the log odds of them reporting financial digital proxy behavior increased by 3.49 and 0.82, respectively. Correspondingly, the log odds of a person reporting medical digital proxy behavior increased by 2.69 and 1.39 with increasing experience with IADL and ADL responsibilities.

### Summary of Findings on Predictors of Reporting Digital Proxy Behavior

In this section, we ran a regression analysis to investigate the predictors of reporting financial and medical digital proxy duties for another adult. The evidence indicated a strong tendency for those who have experience assisting others with IADL and ADL to also report undertaking digital financial and medical responsibilities. Further, our results found *age*, *gender*, and *education* as common nonmodifiable factors significantly influencing a person reporting financial and medical digital proxy duties. We noted additionally that after age 60, female respondents were more likely to report being digital proxies. A person with a higher *income* bracket was more likely to have taken up financial digital proxy duties, though *income* played no role in predicting medical digital proxies. Nevertheless, most proxies were in the household income bracket that included the national average household income or more. A person from an underrepresented ethnic group was less likely to take up medical proxy duties.

### Behaviors of Digital Proxies

**Figure 12.**
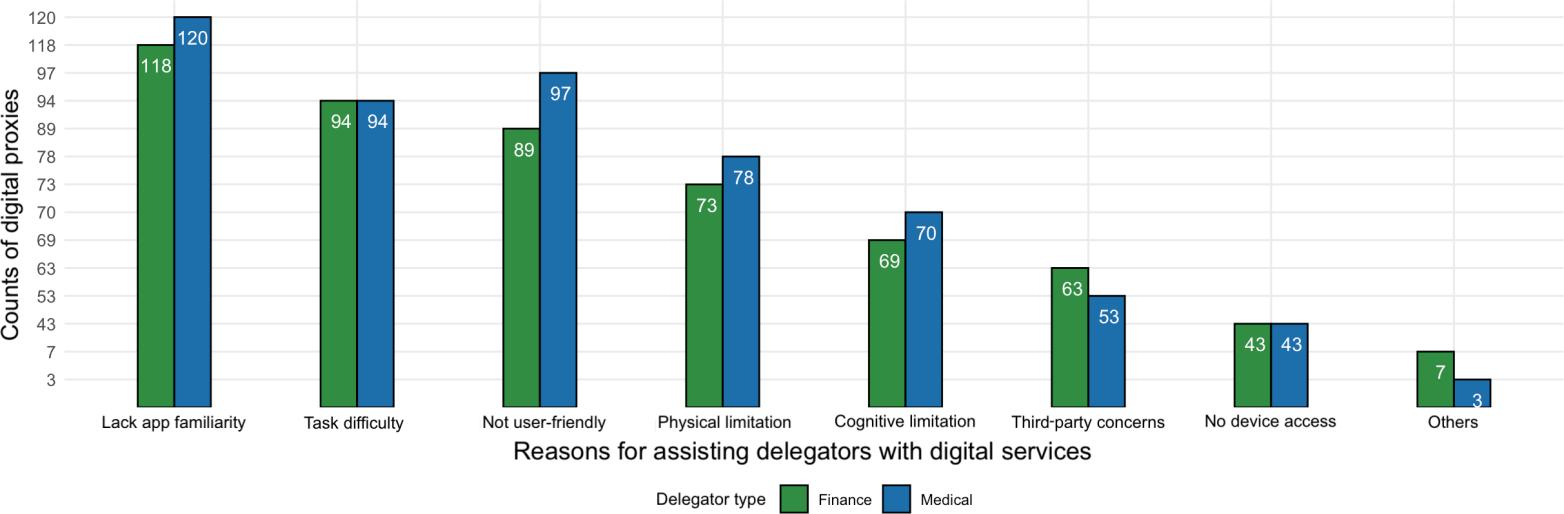
Count of reasons that digital proxies reported that required them to manage digital accounts for other adults, showing a predominance of usability-linked reasons over cognitive and physical limitations. Refer to the Survey Design section for the full questions.

Respondents who identified themselves as digital proxies were asked to choose the most common reasons they had to manage accounts for others. Participants could choose more than one option. We note that across financial and medical digital proxies, the adult’s lack of familiarity with an app (finance n=118; medical n=120), perceived task difficulty (finance n=94; medical n=94), and low usability (finance n=89; medical n=97) were the main reported reasons for the proxy behavior (Figure 12). We also note that less modifiable reasons, such as physical or cognitive limitations of the account owner, were present but not the top reported reasons.

**Figure 13.**
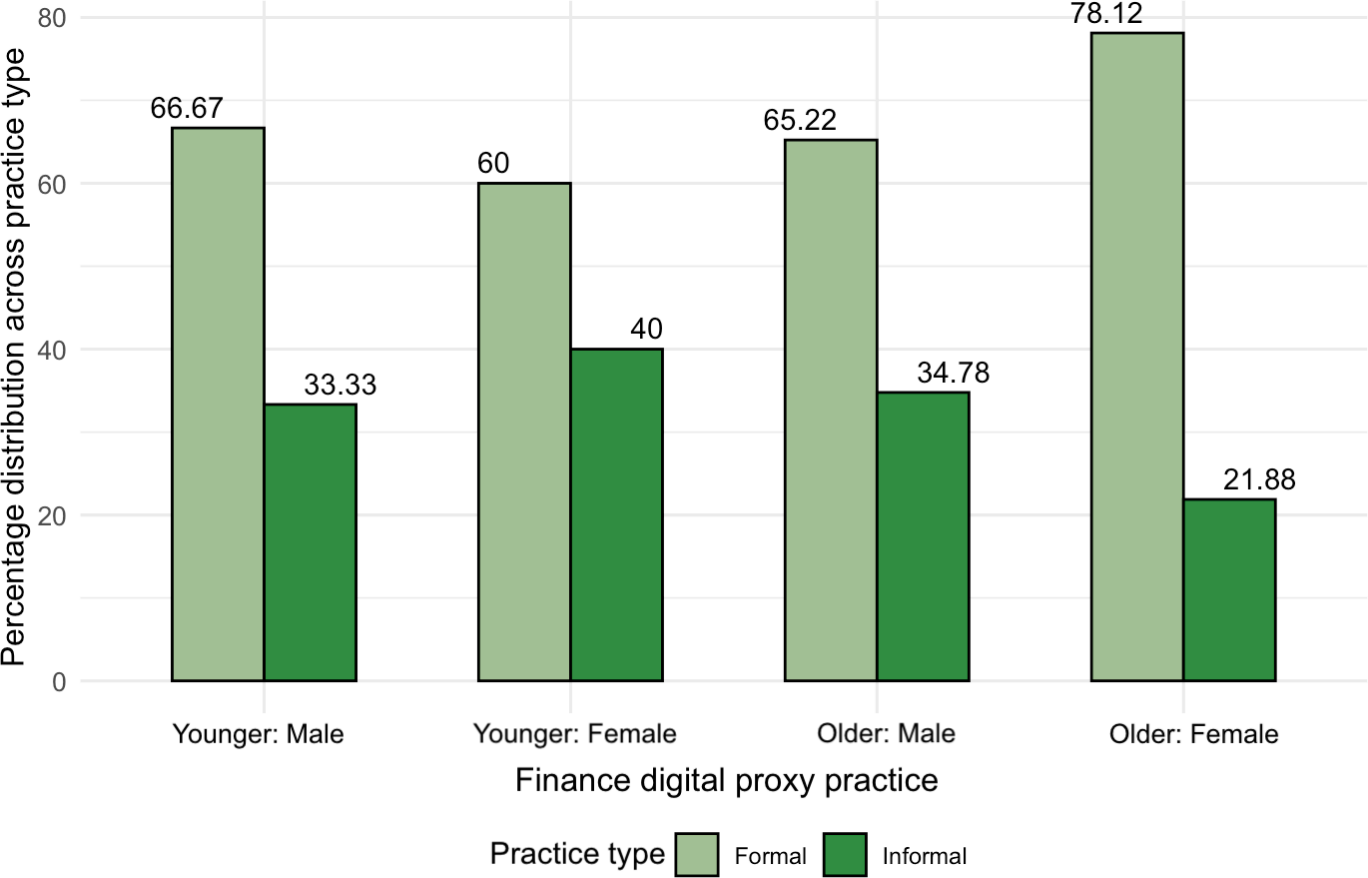
Distribution among all financial digital proxies (n=261), normalized across practice type, showing a higher percentage of formal account usage by female proxies aged 60 years and above.

With these challenges in mind, our analysis now turns to the usage practices of informal digital proxies (who reported access to the delegators’ accounts without having proxy accounts). Recall that we observed an unanticipated reversed effect of increasing participation among female digital proxies above 60. We used this split to further investigate the profiles of digital proxies, and our results yielded a remarkable and intriguing tendency among older female respondents to more often report formal digital proxy practices. As per Figures13  14, the ratio of formal versus informal practices was highest for older females (about 3:1), which goes against the general trend of about 2:1.

**Figure 14.**
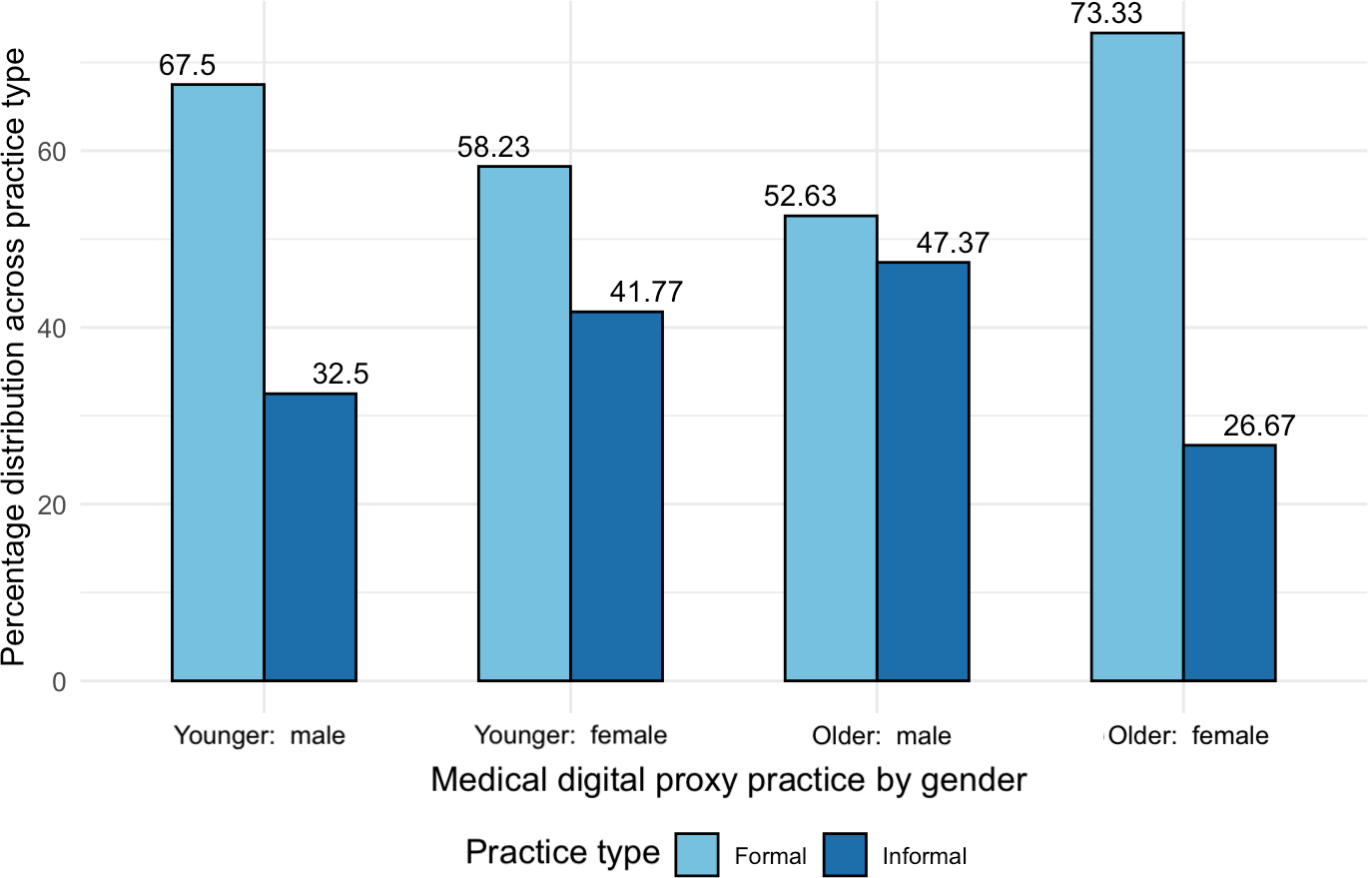
Distribution among medical digital proxies (n=248), normalized across practice type, showing a higher percentage of formal account usage by female proxies aged 60 years and above.

### Informal Proxies’ Login Methods

In what follows, we examine the behaviors of all informal proxies (n=146). In this section, we examine how informal proxies, who make up 22.2% of the total sample, helped their delegators with *logging into* digital accounts.

**Figure 15.**
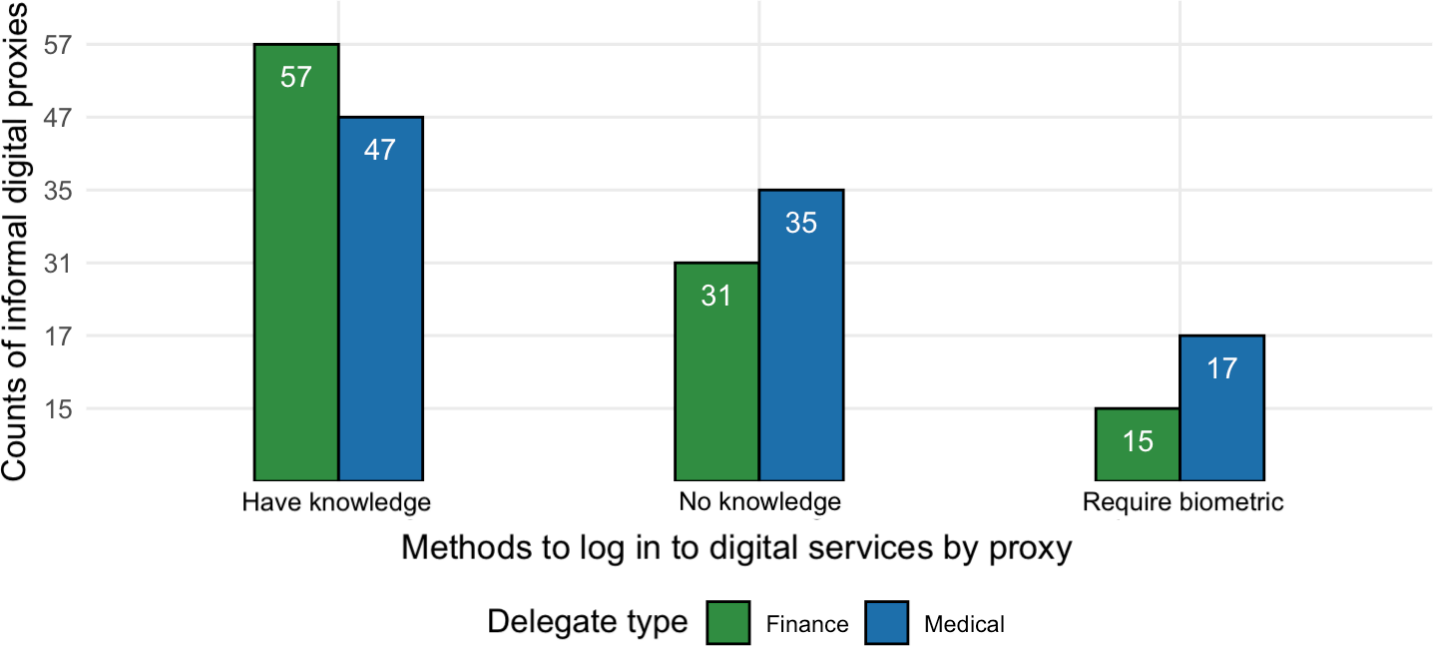
Counts of reported methods of logging in to digital accounts by informal proxies (n=146).

We asked about 3 login methods and report them in Figure 15. Participants could select more than one option, relabeled in the following order: (1) I know the username and password for their online account(s) – *have knowledge*; (2) the adult logs in with a username and password and I help them (I don’t know the username and password) – *no knowledge*; and (3) the adult uses their fingerprint (or other biometrics) to log in *– require biometrics*.

We can see that for both financial and medical informal proxies, more than 5 in 10 informal proxies reported knowing both the username and password of the delegator, while about 3 in 10 informal proxies reported that they have assisted the delegator to log in without knowing their credentials. Finally, about 2 in 10 mentioned that the delegator used biometrics to log in. In other words, about half of informal proxies (or roughly 10% of the total sample) reported having had knowledge of the delegator’s credentials, which potentially may lead to a high risk of misuse.

### Help With Using Digital Accounts

**Figure 16.**
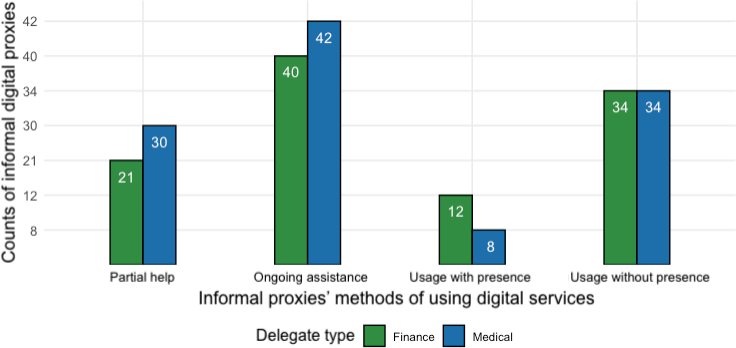
Count of reported methods of using digital accounts by informal proxies (n=146).

In this section, we examine how informal proxies have helped other adults with *using* their digital accounts, as reported in Figure 16. Participants could choose more than one of the following options, relabeled in the following order: (1) I offer some help, but the person does it mostly independently – *partial help*; (2) I provide ongoing assistance while next to the person – *ongoing assistance*; (3) I use the person’s account on their behalf, even when they are not present – *usage without presence*; and (4) I use the person’s account on their behalf, while they are present – *usage with presence*.

We report the distribution of methods practiced by informal proxies when using delegators’ accounts in Figure 16. Most of the informal proxies provided some form of assistance while being beside the delegator. However, about one third (n=34) of informal proxies or roughly 7% of the entire sample reported using the account on their behalf without the presence of the delegator.

### Qualitative Insights

Each section of the survey had an open-ended question asking for other reasons for helping, other methods of logging in, and other methods of helping with using accounts. However, there were only a handful (n=3) of responses, and these did not offer any further insight into the survey data. Hence, we do not report any findings here.

## Discussion

### Principal Findings

This study aimed to quantify the predictors of digital proxies in financial and medical services. We found that nearly half of all respondents had acted as digital proxies, with most performing both medical and financial proxy roles. Digital proxies were typically younger, male, and better educated, and physical caregiving was strongly associated with proxy behavior.

Our findings align with prior qualitative studies suggesting that digital caregiving tasks are often delegated to younger [21] and male [24] tech-savvy individuals. Notably, the predominance of male proxies contrasts with traditional caregiving trends that show women as primary caregivers [2]. Financial proxies were more likely to have higher incomes and less frequent physical caregiving duties, likely due to the remote nature of financial management. Meanwhile, medical proxies were more closely linked with frequent hands-on care. The different predictors and patterns across domains consistent with previous literature [47,48] suggested distinct forms of engagement and responsibility in digital proxying. One critical concern emerging from our data is the large proportion of informal digital proxies—approximately one-third—which may mean that there are millions of individuals in the United States potentially using accounts without proper authorization. This poses risks, particularly in financial services, where informal access may enable elder financial abuse. Although usability barriers are linked to informal delegation, simply introducing more formal mechanisms may not be sufficient. Drawing from the Routine Activities Theory [49,50] and system security literature [3,29,51,52], we argue that effective proxy systems should integrate monitoring, transparency, and capacity-assessing features. Legacy contacts and multiproxy support systems may offer paths forward. The data support recent calls for formal access tools [7] that protect care recipients while accommodating caregiving realities.

### Limitations

This study relies on self-reported data, which is subject to recall bias and social desirability effects, potentially underestimating the prevalence of informal proxying. Additionally, our models were not validated with external datasets, and we did not explore the full range of abuse scenarios or remote care dynamics. Future research should examine these gaps using more diverse and representative samples, explore the motivations of abusers, and evaluate how ongoing access to another adult’s digital accounts might predict harmful behavior.

### Conclusion

Digital proxying is a widespread but understudied caregiving activity with distinct patterns across financial and medical domains. Our findings highlight the growing reliance on younger, educated caregivers to manage digital responsibilities, often without formal authorization. As caregiving demands rise alongside aging populations, policymakers and service designers must develop systems that enable formal proxy access while safeguarding against misuse. Inclusive design, user education, and built-in gatekeeping mechanisms will be critical to balance caregiver support and care recipient autonomy in digital contexts.

## Supplementary material

10.2196/69806 Multimedia Appendix 1 Nationally representative survey sample.

10.2196/69806 Checklist 1 CHERRIES checklist.

## Abbreviations

ADL: Activities of Daily Living
CHERRIES: Checklist for Reporting Results of Internet E-Surveys
IADL: Instrumental Activities of Daily Living
