# Supplementary material for: The Prevalence and Predictors of Digital Proxy Behavior in the United States: Cross-Sectional Survey Study
Source: J Med Internet Res. 2025 Aug 18;27:e69806. doi: 10.2196/69806 (PMC12360674; doi:10.2196/69806)
Supplement: Multimedia Appendix 1 [file jmir-v27-e69806-s001.docx]

NATIONALLY-REPRESENTATIVE SURVEY SAMPLE

Table 7. Dataset is a nationally representative sample, as reported by the US Census Bureau census data from 2020 ^45^.

| Factor | U.S. census % | User Study % | Factor | U.S. census % | User Study % |
| --- | --- | --- | --- | --- | --- |
| Income |  |  | Ethnicity |  |  |
| <$30*𝑘* | 25 | 30 | White | 66.9 | 65 |
| $30*𝑘*-$49*𝑘* | 11.3 | 22 | Black | 12 | 16 |
| $50*𝑘*-$99*𝑘* | 29.7 | 30 | Native | 0.8 | 1 |
| $100*𝑘*-$149*𝑘* | 16.3 | 12 | Asian | 5.1 | 5 |
| $150*𝑘*-$199*𝑘* | 7.9 | 4 | His/Lat | 14.3 | 6 |
| >$200*𝑘* | 9.8 | 2 | Other/Pacific Islander | 1 | 7 |
| Education |  |  | Age |  |  |
| < High School | 12 | 4 | 21-24 | 9.4  (includes ages 18-20) | 8  (excludes ages 18-20) |
| High School | 35 | 50 | 25-29 | 4.7 | 7 |
| Degree & above | 53 | 46 | 30-34  35-39  40-44 | 14.8 7.4  - | 12  10  10 |
| Gender |  |  |  |  |  |
| Male | 49.1 | 49 |  |  |  |
| Female | 50.9 | 51 | 45-49  50-54  55-59 | 1.9  4.7  12.1 | 10  8  7 |
| Housing |  |  |  |  |  |
| Single family unit | 69.3 | 66 |  |  |  |
| 2 or more unit | 22.5 | 27 | 60-64 | 11 | 8 |
| Mobile/Trailer homes | 5.2 | 7 | 65-69 | 5.2 | 10 |
|  |  |  | > 70 | 9.3 | 10 |
